# Effect of Hydration Temperature Rise Inhibitor on the Temperature Rise of Concrete and Its Mechanism

**DOI:** 10.3390/ma16082992

**Published:** 2023-04-10

**Authors:** Tian Liang, Penghui Luo, Zhongyang Mao, Xiaojun Huang, Min Deng, Mingshu Tang

**Affiliations:** 1 College of Materials Science and Engineering, Nanjing Tech University, Nanjing 211800, China; 202061203173@njtech.edu.cn (T.L.); dengmin@njtech.edu.cn (M.D.); 2 State Key Laboratory of Materials-Oriented Chemical Engineering, Nanjing 211800, China

**Keywords:** hydration heat inhibitor, heat of hydration, concrete, degree of hydration

## Abstract

The rapid drop in internal temperature of mass concrete can readily lead to temperature cracks. Hydration heat inhibitors reduce the risk of concrete cracking by reducing the temperature during the hydration heating phase of cement-based material but may reduce the early strength of the cement-based material. Therefore, in this paper, the influence of commercially available hydration temperature rise inhibitors on concrete temperature rise is studied from the aspects of macroscopic performance and microstructure characteristics, and their mechanism of action is analyzed. A fixed mix ratio of 64% cement, 20% fly ash, 8% mineral powder and 8% magnesium oxide was used. The variable was different admixtures of hydration temperature rise inhibitors at 0%, 0.5%, 1.0% and 1.5% of the total cement-based materials. The results showed that the hydration temperature rise inhibitors significantly reduced the early compressive strength of concrete at 3 d, and the greater the amount of hydration temperature rise inhibitors, the more obvious the decrease in concrete strength. With the increase in age, the influence of hydration temperature rise inhibitor on the compressive strength of concrete gradually decreased, and the decrease in compressive strength at 7 d was less than that at 3 d. At 28 d, the compressive strength of the hydration temperature rise inhibitor was about 90% in the blank group. XRD and TG confirmed that hydration temperature rise inhibitors delay early hydration of cement. SEM showed that hydration temperature rise inhibitors delayed the hydration of Mg(OH)_2_.

## 1. Introduction

After the mass concrete is poured, with the cement hydration and heat release, the internal temperature of the mass concrete will change sharply. With the increase in curing time, the strength of concrete increases [1], and the structure is constrained (foundation constraint, new and old concrete contact surface constraint, etc.). Uneven temperature distribution and different constraints within concrete will lead to large temperature stress [2,3]. Once the temperature stress exceeds the permissible tensile strength of concrete, temperature cracks will occur [4,5]. This not only affects the function of the building structure and reduces its stiffness but also affects the durability of the concrete. It has a great negative effect on the mechanical properties and application properties of concrete. Studies show that, with the increase in temperature, the hydration heat release rate of C_3_S accelerates significantly [6]. Therefore, the commonly used measures to control the heat of hydration include using low-heat cement, increasing aggregate content during mixing, controlling the concrete placement temperature and cooling with water [7]. In addition, concrete admixture is also an effective means to improve the thermodynamic properties of concrete, and this method has been widely used in engineering [8,9]. From then on, the choice of concrete admixture is the key to solve the problem of concrete cracking. Hydration heat inhibitors [10,11,12] is a new type of concrete admixture, prepared by acid hydrolysis of corn starch. It is used to solve the problem of temperature cracking caused by excessive thermal stress in mass concrete [13,14]. Studies have shown that adding hydration heat inhibitors to concrete can effectively reduce the heat release rate of cement in the hydration acceleration stage without affecting the total heat release of hydration. That is, temperature rise inhibitors can regulate the hydration process and reduce the heat release rate of hydration [15,16]. The main components of existing hydration heat inhibitors are hydroxyl carboxylic acid esters, starch or dextrin and its derivatives, active heat-absorbing salts, etc. In addition, the hydration heat inhibitor can be modified to form a new type of hydration heat inhibitor. Relevant studies [15] have shown that the addition of hydration heat inhibitors has a significant impact on the nucleation of C-S-H, which is mainly manifested as reducing the main peak of hydration reaction. Hydration heat inhibitors cement hydration mainly by regulating the induction and acceleration time of C_3_S hydration, and the hydration heat inhibitors’ effect on the early stage of cement hydration is greater than that in the later stage because, once the nucleus is formed, the growth of C-S-H will be very stable and will not be affected by hydration heat inhibitors. Many domestic and foreign scholars have studied the effect of hydration heat inhibitors on cement hydration [17] and concrete hydration [11]. These studies basically focus on the effects of hydration heat inhibitors on pre-hydration or post-hydration, and they often ignore the coordination between the two. Specifically, hydration heat inhibitors can reduce the early strength of concrete, delay the concrete setting time, etc. This limits its application in engineering cast-in-place structural concrete. Therefore, it is necessary to develop a hydration heat inhibitor that has less influence on the setting time and early strength of concrete.

In this paper, the effects of adding different hydration heat inhibitor dosages on cement concrete were studied from macroscopic properties and microscopic structure characteristics. Macroscopic performance shows that the addition of hydration heat inhibitor has the effect of delaying cement hydration and can relatively reduce the internal temperature rise of concrete and delay the temperature rise. Based on the characteristics of microstructure, the mechanism of hydration heat inhibitor on concrete was analyzed from the aspects of phase composition, pore structure and morphology.

## 2. Materials and Methods

### 2.1. Materials

The cement selected for the experiment was Nanjing Onoda PII 52.5 silicate cement; the fly ash was secondary ash; the mineral powder was S95 grade mineral powder; the expansion agent was MgO expansion agent produced by Jiangsu Subote New Materials Joint Stock Company (Zhenjiang, China); the fine aggregate was river sand with a fineness modulus of 2.7; the coarse aggregate was 5–31.5 mm continuously graded crushed stone; the water reducing agent was poly(carboxylic acid) high-performance water reducing agent produced by Jiangsu Subote New Materials Joint Stock Company. The water reducing agent is poly-carboxylic acid high-performance water reducing agent produced by Jiangsu Subote New Materials Co. (Nanjing, China) The water reducing agent is the high-performance water reducing agent of polycarboxylic acid produced by Jiangsu Subote New Materials Co. Hydration heat inhibitor is the product of Jiangsu Subote New Materials Co., hereinafter referred to as SBT.

**Figure 1:**
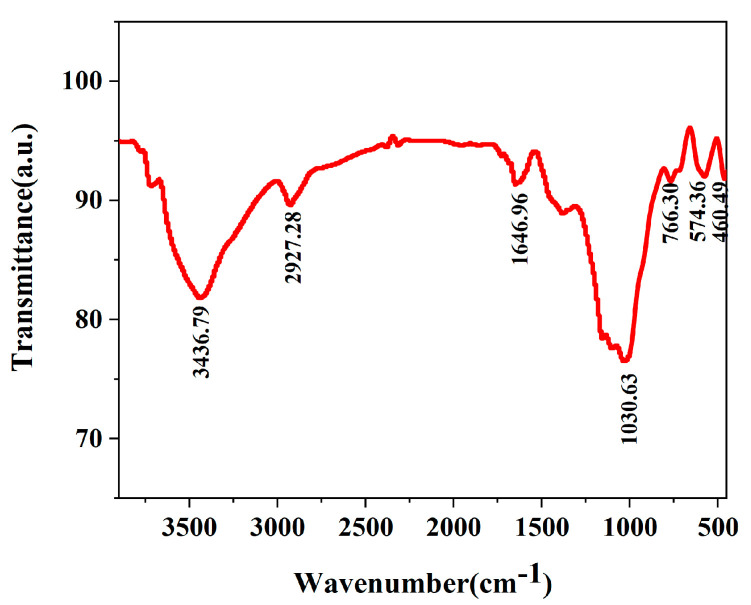
FTIR spectra of SBT with a resolution of 0.4 cm^−1^.

The chemical composition of the raw material is shown in Table 1. The functional groups test of the SBT is shown in Figure 1. In Figure 1, the functional group test of the SBT is performed using Fourier transform infrared spectroscopy (Bruker Equinox 55, resolution 0.4 cm^−1^). The absorption peaks of 3436.79 cm^−1^, 2927.68 cm^−1^, 1030.63 cm^−1^ and 766.30 cm^−1^ correspond to the tensile vibrations of hydroxyl (-OH), C-H, C-O and C-O-C, respectively.

### 2.2. Sample Preparation

In order to study the effect of hydration temperature rise inhibitor on concrete temperature rise and its mechanism, first of all, the concrete fit used in the experiment is shown in Table 2. The dosage of hydration temperature rise inhibitor was 0%, 0.5%, 1.0% and 1.5% of the total dosage of cementing material, respectively. Then, the specific experimental method is to stir these ingredients under dry conditions, and then add quantitative water to continue to stir evenly. The fully mixed slurry is placed in a mold of a specific size for the internal temperature rise of concrete and the 3 d, 7 d and 28 d compressive strength of concrete. In addition, without considering the addition of coarse and fine aggregates, after the raw materials are fully stirred, part of the slurry is put into a specific mold for a medium to 3 d, 7 d and 28 d age, which is used for micro-tests, such as hydration heat and thermogravimetry.

### 2.3. Methods

#### 2.3.1. Macroscopic Experimental Methods

The macroscopic experimental methods in this paper include hydration heat test, concrete temperature rise test and mechanical test. The heat of hydration test adopts dissolution method, that is, isothermal calorimeter. Firstly, a certain amount of fresh cement paste was placed in the test tube, and then the data were monitored for 3 days. Finally, the hydration heat and cumulative heat release data of the cement paste were obtained. The temperature rise test of concrete uses a thermometer to measure, the concrete is mixed into a fixed mold and then the thermometer is added to record the change in its internal temperature in real time. The insulation device uses an iron bucket with a bottom diameter of 300 mm and a height of 300 mm. The bottom and outside of the bucket are wrapped with 5 cm thick rubber and plastic insulated cotton. After the wrapping is completed, the whole bucket is put into the insulation bucket of the same size. The thermometer is inserted into the center of the concrete, and the thermometer connecting module records the internal temperature in real time. The mechanical properties were tested by universal pressure testing machine. In each age, 3 specimens were used to test the compressive strength, and their average values were recorded.

#### 2.3.2. Microscopic Experimental Analysis

In this paper, the microscopic characterization techniques include XRD, TG, MIP, SEM, etc. At the end of the mechanical test, appropriate amount of the broken sample was soaked in anhydrous ethanol to stop hydration for a week, and then dried in vacuum at 80 °C for 2 days. After taking out the sample, it was used for SEM. The powder sample is then prepared, and the sample is ground into powder and passed through a 200-mesh screen to prepare the sample for XRD and TG analysis. The phase composition of the sample was characterized by XRD and TG. XRD analysis was used to observe the difference in the composition of crystalline mineral phase in the hydration products. The quantitative analysis of Mg(OH)_2_ in cement slurry was performed by TG analysis. Obtained by STA 409PC Luxx at 10 °C/min from 30 °C to 1000 °C in a nitrogen atmosphere. The pore structure was characterized by mercury porosity method in the pressure range of 10–100 kPa, corresponding to pore size range of 1.7–300 nm. The microstructure of the concrete was characterized by SEM, in which the dry powder required prior spraying of gold, and scanned using Merlin Compact scanning electron microscopy.

## 3. Results and Discussion

### 3.1. Macroscopic Properties

#### 3.1.1. Mechanical Property

**Figure 2:**
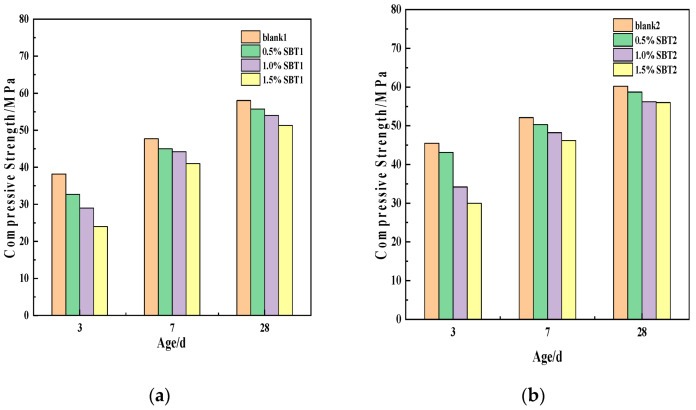
Compressive strength of concrete specimens at different ages. (**a**) is the compressive strength of concrete specimen under normal temperature curing; (**b**) is the compressive strength of concrete specimen under variable-temperature curing.

As shown in Figure 2a, the compressive strength of concrete increases gradually with the increase in age. In the concrete mixed with SBT, the compressive strength decreases with the increase in the amount of SBT. In the first 3 days, it decreased significantly with the increase in variables. However, the reduction degree of compressive strength at 28 days decreased compared with that at 3 days. For example, the compressive strength of 0.5%SBT1 and 1.5%SBT1 was 14.4% and 37.2% lower than blank at 3 d, and 4.0% and 11.6% lower than blank at 28 d, respectively. The reason may be that SBT hindered the nucleation and growth of C-S-H in the early stage but did not affect the degree of hydration in the later stage [10].

Figure 2b shows the compressive strength of concrete specimens at different ages when they are placed in a curing box at variable temperature. The compressive strength of concrete increases under variable temperature conditions. For example, the compressive strength of 0.5%SBT2 and 1.5%SBT2 at 3 d increases by 24.1% and 20%, respectively, compared with that at normal temperature. This is because the higher the temperature, the higher the internal hydration rate and the higher the strength of the concrete.

#### 3.1.2. Temperature Rise Experiment

**Figure 3:**
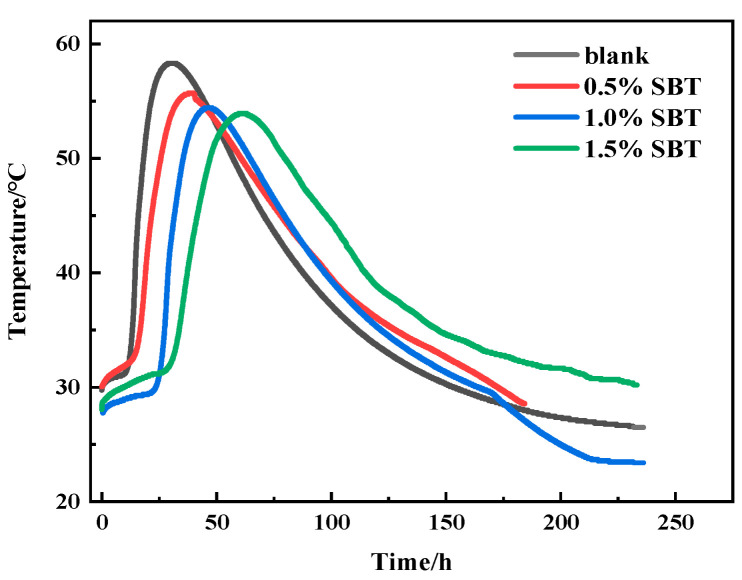
Internal temperature of concrete specimen.

Figure 3 shows the internal temperature changes in concrete specimens after adding different amounts of SBT. As shown in the figure, with the increase in the amount of SBT, the temperature inside the concrete began to rise relatively late, and its peak temperature also decreased relatively. For example, the peak temperature of 0.5%SBT is 10.6 h later than blank, and the peak temperature is 4.5% lower. Further, 1.5%SBT is delayed for 32.3 h and the peak temperature is reduced by 7.5%. Equipment error is negligible. The reason is that the addition of SBT will hinder the nucleation and growth of C-H-S inside the concrete, thus reducing the hydration rate inside the concrete, resulting in a smaller internal temperature, resulting in a lower temperature rise inside the concrete. This is consistent with the conclusion above; that is, the temperature rise of concrete is slow, the peak temperature is low and the compressive strength of concrete specimens decreases.

#### 3.1.3. Hydration Heat

**Figure 4:**
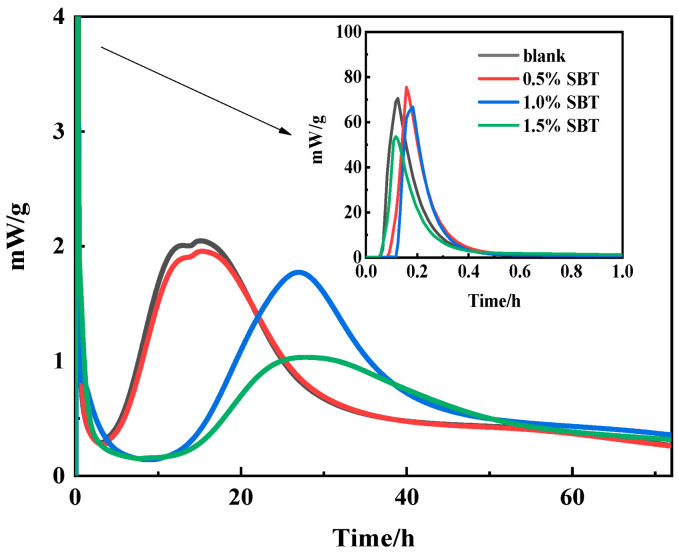
Heat map of hydration of cement paste.

Figure 4 shows the heat of hydration of the cement slurry in 3 d. According to previous conclusions, MEA can enhance the release of hydration heat, which is manifested as advanced induction period and increase in heat flow [18]. When SBT is added, the induction period and heat release peak are delayed relatively. With the increase in SBT incorporation, the early internal heat release decreased gradually. Therefore, for cement slurry, the addition of SBT will inhibit the internal hydration reaction, thus reducing the internal temperature rise and temperature peak. By extension, it can be concluded that the addition of hydration heat inhibitors can effectively reduce the temperature difference between inside and outside of concrete in the early stage, thus reducing the risk of concrete cracking.

### 3.2. Microstructural Characteristics

#### 3.2.1. XRD

**Figure 5:**
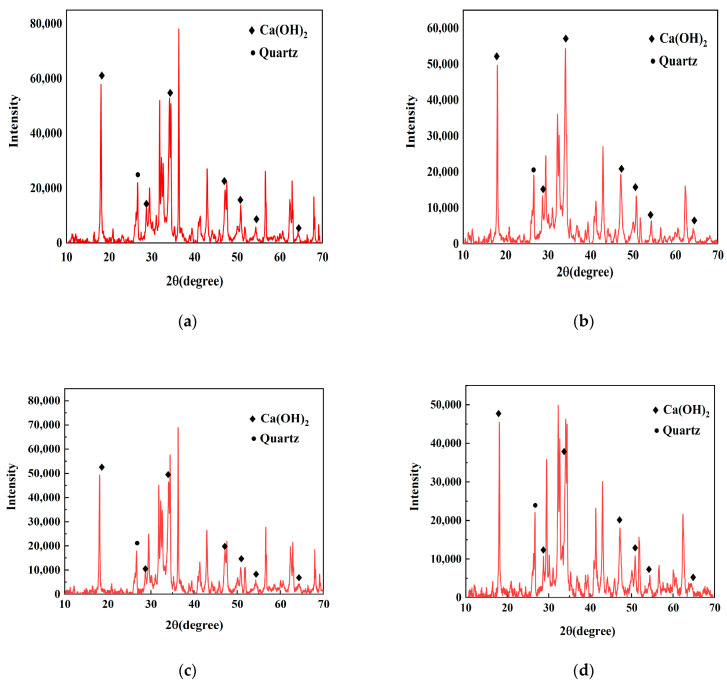
XRD pattern of cement slurry, where (**a**) is blank reference group; (**b**) the hydration temperature rise inhibitor group containing 0.5%SBT; (**c**) is the dosage of 1.0%SBT hydration temperature rise inhibitor group; (**d**) is the dosage of 1.5%SBT hydration temperature rise inhibitor group.

As can be seen from Figure 5, compared with the base group, the Ca(OH)_2_ diffraction peak strength of the cement slurry mixed with different amounts of SBT is lower than that of the base group, the Ca(OH)_2_ diffraction peak strength of the cement slurry mixed with 0.5%SBT hydration temperature rise inhibitor is the strongest among the three groups and the calcium hydroxide diffraction peak strength of the cement slurry mixed with 1.5%SBT is the lowest. The principle of cement hydration degree was tested by referring to CH quantitative test method. It is concluded that different amounts of SBT can effectively delay cement hydration, and, with the increase in its dosage, its inhibition effect will be better.

#### 3.2.2. TG

**Figure 6:**
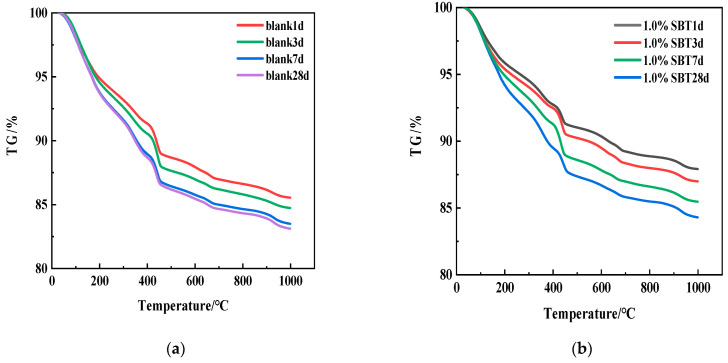
TG diagrams of cement slurry at 1 d, 3 d, 7 d and 28 d. (**a**) The TG curves of the blank group at 4 instars; (**b**) is the TG curve of 4 instars of hydration temperature rise inhibitor containing 1.0%SBT.

**Figure 7:**
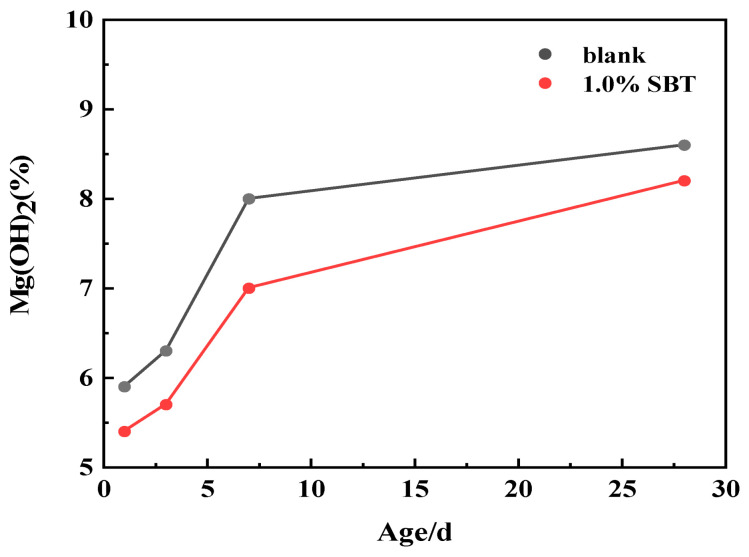
The degree of hydration of MgO at each age.

Figure 6 shows the TG curves of the sample containing 1.0% SBT hydration heat inhibitor at 1, 3, 7 and 28 days. It corresponds to the dehydration decomposition of hydration products C-S-H, C-A-H, AFm, AFt, etc. [19]. Mg(OH)_2_, Ca(OH)_2_ and CaCO_3_ will thermal decompose at peak absorption temperatures of 330–420 °C, 420–500 °C and 600–750 °C, respectively [20,21]. The relevant equations are shown in Equations (1)–(3). Table 3 shows the mass scores of blank and 1.0%SBT at different temperatures. Meanwhile, Mg(OH)_2_ content can be calculated according to Equation (4) and Table 3, and the results are shown in Figure 7.

Mg(OH)_2_→MgO + H_2_O, (1)

Ca(OH)_2_→CaO + H_2_O (2)

CaCO_3_→CaO + CO_2_ (3)

(4) $${\mathrm{WMg}(\mathrm{OH})}_{2}\;=\Delta W_{330–420{}^{\circ}C}\frac{\mathrm{MMg}{\left(\mathrm{OH}\right)}_{2}}{{\mathrm{MH}}_{2}O}$$

where WMg(OH)_2_ is the mass fraction(%) of Mg(OH)_2_; MMg(OH)_2_ is the molar mass of Mg(OH)_2_ (g/mol); ΔW_330–420 °C_ represents the mass loss fraction(%) in the 330–420 °C temperature range.

The degree of hydration of MgO can be discussed through quantitative analysis in the figure. As can be seen from Figure 7, Mg(OH)_2_ of 1.0%SBT decreased in each age compared with blank, but the total amount still increased with age. The decrease in Mg(OH)_2_ after the addition of SBT was due to the fact that SBT could adsorb on the surface of MEA and prevent the hydration of MgO, thus leading to the decrease in Mg(OH)_2_. On the other hand, it is also possible that Mg(OH)_2_ reacts with SiO_2_ to form M-S-H and then Ca(OH)_2_, which also relatively reduces the degree of hydration of Mg(OH)_2_.

#### 3.2.3. MIP

**Figure 8:**
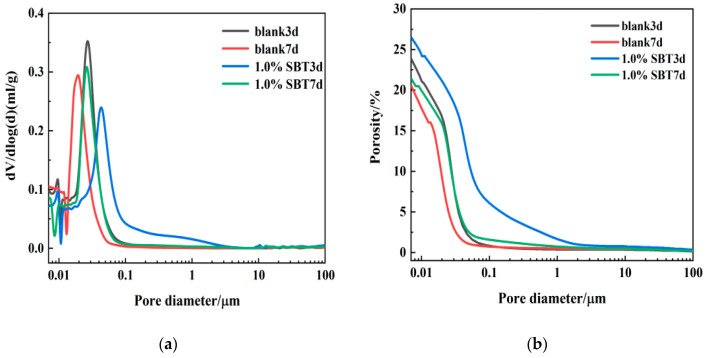
3 d and 7 d mercury injection maps of blank cement paste group and 1.0%SBT. (**a**) is the aperture distribution diagram of each specimen; (**b**) is the cumulative aperture distribution diagram of each specimen.

Another important parameter to reflect the microstructure is to observe the pore structure. It can be obtained from the aperture distribution and cumulative aperture distribution as shown in Figure 8. The pore size of blank group and 1.0%SBT group was mainly distributed between 10 nm and 100 nm. It can be seen from 3 d and 7 d data that the primary aperture of blank group and 1.0%SBT group at 7 d is larger than that of 3 d group. It can be seen from the figure that the main pore diameter of the sample after adding 1.0%SBT is larger than that of the blank group. In addition, it can be seen from Figure 8b that the porosity values from large to small are 1.0%SBT3d, blank3d, 1.0%SBT7d, blank7d. The microstructure of cement slurry without hydration heat inhibitor is denser and its corresponding compressive strength is relatively higher.

#### 3.2.4. SEM

**Figure 9:**
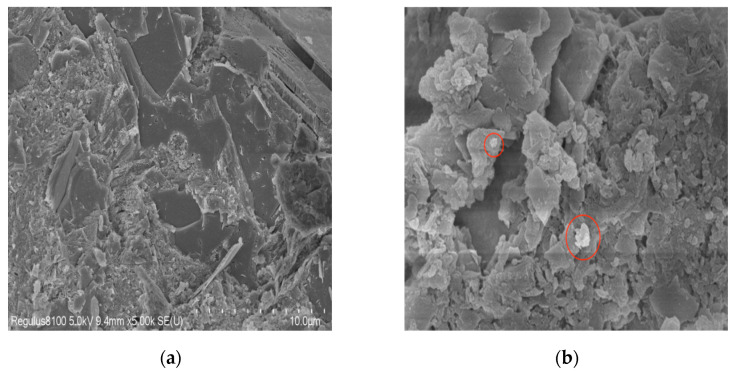
SEM image of 3 d concrete specimen. (**a**) SEM diagram of concrete specimen without water-added temperature rise inhibitor 3 d; (**b**) SEM diagram of concrete specimen doped with 1.0%SBT hydration temperature rise inhibitor.

In order to better understand the differences in mechanical properties between blank group and 1.0%SBT group in early stage, the concrete specimens were photographed by electron microscopy. Figure 9 shows SEM observations of concrete of corresponding age. On the whole, the concrete structure is relatively loose, and there are obvious voids and holes in the structure. As can be seen from Figure 9a, hydration products, such as acicular ettringite, hexagonal crystalline calcium hydroxide and flocculent calcium silicate hydrate, exist in the surface topography of concrete, and no other special substances are observed. In addition to the above substances, some white crystals circled as shown in the picture are also observed in Figure 9b, which are partially incomplete SBT residue and partially MgO, indicating that the hydration heat inhibitor delays the hydration process of cement and also reduces the degree of MgO hydration.

## 4. Conclusions

In this paper, the effect and mechanism of hydration heat inhibitors on the temperature rise of concrete are studied systematically from two aspects of macroscopic property and microscopic structure. According to the above experimental results, the following conclusions can be drawn.

1. The compressive strength of concrete with hydration heat inhibitors decreased by 14.4–37.2% at 3 d. This indicates that the addition of heat of a hydration inhibitor can significantly reduce the early compressive strength of concrete. It was reduced by about 10% at 28 d to show that it had little effect on the concrete in the later stage.
2. It can be seen from the internal temperature rise diagram and hydration heat map of concrete that hydration heat inhibitor delays the time of concrete reaching the temperature peak by 10–30 h. Adding hydration heat inhibitors significantly reduces the heat flow and heat release rate of cement, prolongs the setting time of cement, inhibits the early hydration process of cement and improves the mechanical properties of cement in the later stage.
3. XRD and TG results show that the hydration heat inhibitor does not stop the hydration process of cement and has no effect on the type and morphology of hydration products. It can only delay the hydration process of cement and reduce the content of magnesium hydroxide and calcium hydroxide in hydration products. Hydration heat inhibitors can delay cement hydration.
4. The results of MIP experimental analysis and SEM image observation show that the addition of hydration heat inhibitors can increase the main pore size and porosity of cement, which is also the result that the hydration heat inhibitor delays the early hydration degree of cement. At the same time, the microstructure of concrete has not changed much. Specifically, the concrete structure is relatively loose, obvious voids and holes can be observed and only part of the MgO and hydration heat inhibitors are not fully reacted. This further indicates that the addition of heat of hydration inhibitor is not conducive to the microstructure densification of cement slurry and delays the degree of hydration of MgO.

## Figures and Tables

**Table 1:** Chemical composition of raw materials/%.

Raw Materials	SiO_2_	Al_2_O_3_	Fe_2_O_3_	CaO	MgO	K_2_O	Na_2_O	SO_3_	Loss
Cement	18.55	3.95	3.41	65.32	1.01	0.72	0.18	2.78	2.88
FA	44.06	42.06	2.91	3.80	0.40	0.49	0.16	0.75	2.48
S95	33.39	11.89	0.63	41.51	8.82	0.53	0.67	/	0.28
MEA	3.87	1.03	0.88	1.98	89.37	0.88	/	0.06	2.38

**Table 2:** Concrete proportioning (kg/m^3^).

Number	Cement	FA	S95	MEA	Sand	Stones	Water	Water Reducing Agent	SBT
Blank	288	90	36	36	700	1100	144	12.6	0
0.5%SBT	288	90	36	36	700	1100	144	12.6	2.25
1.0%SBT	288	90	36	36	700	1100	144	12.6	4.5
1.5%SBT	288	90	36	36	700	1100	144	12.6	6.25

**Table 3:** The mass fraction of each sample at different temperatures.

Temperature (°C)		30	330	420	500	600	750
Blank	1 d	100	92.62	90.92	88.67	87.93	86.77
	3 d	100	91.97	90.15	87.63	86.96	85.98
	7 d	100	90.83	88.57	86.42	85.75	84.79
	28 d	100	90.68	88.25	86.16	85.44	84.46
1.0%SBT	1 d	100	92.44	92.44	91.01	90.35	89.22
	3 d	100	92.08	92.08	90.23	89.49	88.11
	7 d	100	90.63	90.63	88.58	87.81	86.96
	28 d	100	89.16	89.16	87.35	86.68	85.62

## Data Availability

The data presented in this study are available on request from the corresponding author.
